# Improved Visualization and Quantification of Net Water Uptake in Recent Small Subcortical Infarcts in the Thalamus Using Computed Tomography

**DOI:** 10.3390/diagnostics13223416

**Published:** 2023-11-09

**Authors:** Felix Schön, Hannes Wahl, Arne Grey, Pawel Krukowski, Angela Müller, Volker Puetz, Jennifer Linn, Daniel P. O. Kaiser

**Affiliations:** 1 Institute and Polyclinic for Diagnostic and Interventional Neuroradiology, Faculty of Medicine and University Hospital Carl Gustav Carus, Technische Universität Dresden, 01307 Dresden, Germany; 2 Institute and Polyclinic for Diagnostic and Interventional Radiology, Faculty of Medicine and University Hospital Carl Gustav Carus, Technische Universität Dresden, 01307 Dresden, Germany; 3 Dresdner Neurovaskuläres Centrum, Faculty of Medicine and University Hospital Carl Gustav Carus, Technische Universität Dresden, 01307 Dresden, Germany; 4 Department of Neurology, Faculty of Medicine and University Hospital Carl Gustav Carus, Technische Universität Dresden, 01307 Dresden, Germany

**Keywords:** recent small subcortical infarcts, net water uptake, computed tomography, thalamus, postprocessing, frequency-selective non-linear blending

## Abstract

Diagnosing recent small subcortical infarcts (RSSIs) via early computed tomography (CT) remains challenging. This study aimed to assess CT attenuation values (Hounsfield Units (HU)) and net water uptake (NWU) in RSSI and explore a postprocessing algorithm’s potential to enhance thalamic RSSI detection. We examined non-contrast CT (NCCT) data from patients with confirmed thalamic RSSI on diffusion-weighted magnetic resonance imaging (DW-MRI) between January 2010 and October 2017. Co-registered DW-MRI and NCCT images enabled HU and NWU quantification in the infarct area compared to unaffected contralateral tissue. Results were categorized based on symptom onset to NCCT timing. Postprocessing using window optimization and frequency-selective non-linear blending (FSNLB) was applied, with interpretations by three blinded Neuroradiologists. The study included 34 patients (median age 70 years [IQR 63–76], 14 women). RSSI exhibited significantly reduced mean CT attenuation compared to unaffected thalamus (29.6 HU (±3.1) vs. 33.3 HU (±2.6); *p* < 0.01). Mean NWU in the infarct area increased from 6.4% (±7.2) at 0–6 h to 16.6% (±8.7) at 24–36 h post-symptom onset. Postprocessed NCCT using these HU values improved sensitivity for RSSI detection from 32% in unprocessed CT to 41% in FSNLB-optimized CT, with specificities ranging from 86% to 95%. In conclusion, CT attenuation values and NWU are discernible in thalamic RSSI up to 36 h post-symptom onset. Postprocessing techniques, particularly window optimization and FSNLB, moderately enhance RSSI detection.

## 1. Introduction

Recent small subcortical infarcts (RSSIs) are the neuroimaging correlate of lacunar strokes, which account for approximately 25% of all ischemic strokes [1,2]. They are defined as small subcortical infarcts with a maximum diameter of 15–20 mm in the territory of one perforation artery, typically located in the basal ganglia, internal capsule, thalamus, brainstem, or subcortical white matter. The etiology of RSSI is usually due to microangiopathic vessel wall changes in cerebral small vessel disease [2,3,4]. Patients may present with different symptoms depending on the anatomical location of the RSSI. Although the mortality is low (approximately 2.5% within the first 30 days), up to one-third of all patients are still dependent on the help of others at one year, demonstrating the importance of early diagnosis and initiation of therapy [5].

Diffusion-weighted magnetic resonance imaging (DW-MRI) is the gold standard for diagnosis of RSSIs [2,6]. However, in most centers, computed tomography (CT) evolved to be the mainstay in acute stroke imaging. Compared with MRI, CT allows faster examination times, has fewer contraindications, and is more widely available. However, only a minority of RSSIs can be diagnosed by multimodal CT within the first three days after symptom onset [7,8,9]. In addition to small infarct size, detection of these infarcts on non-contrast CT (NCCT) is limited by poor visual delineation of the ischemic tissue compared with non-infarcted brain tissue. Improving RSSI detection on NCCT is, therefore, an important objective.

In ischemic infarct, a critical perfusion decline results in the formation of ionic edema and increased net water uptake (NWU) into the ischemic tissue. The increased NWU can be detected by decreased CT attenuation measured in Hounsfield Units (HU) [10,11].

Data on NWU and CT attenuation of RSSIs have not been published in the current literature. The knowledge about the CT characteristics may help to increase the detection rates of these infarcts, for example, by adopting postprocessing and imaging feature analysis methods, such as selective windowing or frequency selective non-linear blending (FSNLB), an algorithm to augment HU in a predefined range. Both methods have been implemented for the diagnosis of middle cerebral artery (MCA) infarcts [12,13]. However, knowledge of the CT attenuation values is required for sufficient transfer of both methods in RSSI detection.

Overall, the thalamus is the second most common location of RSSIs [14]. Due to the pronounced neuroanatomical connections, the infarcted thalamus can cause many neurological deficits. Typically, these include sensory and motor deficits, as well as disturbances of consciousness, but large cortical infarcts can also be mimicked [15,16]. Thalamic RSSIs thus represent a particular challenge for treating physicians in everyday clinical practice.

Therefore, we aimed to determine CT attenuation and NWU in the RSSI of the thalamus, as it is a frequent location of RSSIs and allows reliable assessment of CT attenuation. Further, we aimed to investigate whether the detection of RSSIs could be increased by NCCT postprocessing using window optimization and FSNLB.

## 2. Materials and Methods

### 2.1. Patients

The present study was approved by the local ethics committee (EK 315082017). The informed consent was waived due to the retrospective nature of the study. The study complied with the Declaration of Helsinki and the Strengthening the Reporting of Observational Studies in Epidemiology (STROBE) statement [17].

Three patient cohorts were defined for the present study: main cohort (patients with RSSI of the thalamus), control cohort 1 (patients with RSSI outside the thalamus), and control cohort 2 (patients without infarction).

We retrospectively screened stroke patients who were admitted to our center between January 2010 and October 2017. We included all patients with acute onset of stroke symptoms who received NCCT with a slice thicknesses ≤ 1.5 mm within 36 h of symptom onset and in whom subsequent DW-MRI as the gold standard of RSSI detection confirmed a single recent, DWI-positive RSSI in the thalamus within 10 days. The DWI-positive RSSI matched the stroke symptoms, showed corresponding T2-weighted hyperintensities, and MRI excluded contralateral thalamus lesions. Further exclusion criteria were (1) multiple or old infarcts, (2) no information on symptom onset or last known well, (3) intracranial hemorrhage, inflammation, and tumor, (4) other than small vessel subtype of stroke etiology [18]. Experienced stroke neurologists assessed the most likely etiology of stroke based on clinical workup and imaging. Based on equivalent criteria, patients with RSSI (control cohort 1) outside the thalamus were included. Patients without infarction had NCCT with slice thicknesses ≤ 1.5 mm due to various medical indications (TIA, epilepsy, trauma, etc.). No acute intracranial pathology was allowed.

We documented baseline characteristics, including age, sex, vascular risk factors, stroke symptoms (National Institutes of Health Stroke Scale, NIHSS score), and administration of intravenous thrombolysis (IVT).

### 2.2. Imaging

We documented imaging characteristics, including time from symptom onset or last known well to NCCT, infarct location, and the extent of white matter lesions (WML) on MRI with the Fazekas scale (0–3, with 0 indicating absent and 3 indicating large confluent areas of WML) [19].

Patients received comprehensive stroke imaging with CT at admission and MRI follow-up, including DWI, FLAIR, T2*-weighted, T2-weighted, and time-of-flight (TOF-) angiography sequences. NCCT scans were acquired with a tube voltage and current of 120 kV and 250, 350, or 370 mAs and a slice thickness of 1.2 mm or 1.5 mm on three different scanners (Somatom Definition AS+, Sensation 16, Sensation 64; Siemens Healthineers, Erlangen, Germany). Image datasets were reconstructed with a soft tissue kernel and a window level/width of 35/60 HU.

We acquired DW-MRI (b value, 0 and 1000 s/mm^2^; slice thickness 3 mm and 5 mm) on 1.5 or 3.0 Tesla scanners (Avanto, Magnetom Vision, Sonata, Verio; Siemens Healthineers, Erlangen, Germany, and Signa HDxt; GE Healthcare, Solingen, Germany).

### 2.3. Co-Registration

**Figure 1:**
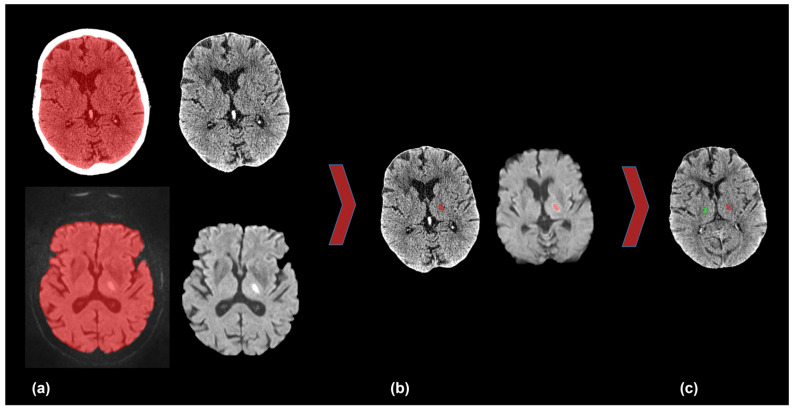
Co-registration of DW-MRI and NCCT. (**a**) Semi-automatic segmentation and brain extraction in NCCT (upper row) and DW-MRI (lower row). (**b**) Co-registration of NCCT and DW-MRI and infarcted area in the left thalamus (red). (**c**) Mirroring the left-sided infarct (red) onto the unaffected contralateral right thalamus (green). DW-MRI = diffusion-weighted magnetic resonance imaging, NCCT = non-contrast computed tomography.

To quantify CT attenuation values of thalamic RSSI, a semi-automatic workflow of brain extraction, co-registration, and segmentation was performed. The aim was to mirror the infarcted area on DW-MRI to the NCCT to collect the CT attenuation values of the infarct and contralateral unaffected thalamus. Figure 1 illustrates the described workflow.

In detail, we exported the NCCT and DW-MRI data to the open-source software ITK-SNAP [20] and performed a threshold-based semi-automatic segmentation of the brain parenchyma followed by co-registration of the NCCT and MRI. Subsequently, we performed semi-automatic segmentation of the infarcted area in the DW-MRI, including the use of “binary erosion” and “fill hole” methods for image postprocessing. The areas of the DW-MRI lesion were shrunk by approximately 10%. We checked and manually corrected all segmentations if necessary. Then, we normalized and transferred the co-registered image data to the MNI 152 space [21,22]. Subsequently, we mirrored the segmentation of the infarcted area to the contralateral thalamus against the midline with the use of an in-house algorithm, again followed by visual verification. Finally, we transformed the mirrored segmentation back to the native NCCT space.

### 2.4. Quantification of Net Water Uptake

We used a standardized procedure to quantify NWU of the thalamic RSSI, as reported elsewhere [10]. Briefly, we measured the mean CT attenuation values of the co-registered infarcted area (HU*_ischemic_*) and of the mirrored area in the contralateral thalamus (HU*_normal_*) on admission to NCCT. We calculated the NWU of the recent lacunar infarcts with the equation:

% NWU = (1 − HU*_ischemic_*/HU*_normal_*) × 100 (1)

Based on the distribution of patients according to symptom onset (or last known well) to NCCT time, we categorized mean HU values and NWU into time windows.

### 2.5. Image Ppostprocessing

NCCT window leveling and FSNLB were performed for image postprocessing. Window level and width were defined as the mean and twice the standard deviation (SD) of the previously acquired CT attenuation values of the infarcted areas.

For FSNLB, an established algorithm, Best Contrast (BC), a client server application (SyngoVia, Research Frontier; Siemens Healthineers, Erlangen, Germany), was used as reported elsewhere [23]. Briefly, using a Fourier transformation, BC converts the attenuation values into a spectrum of high (containing image noise) and low (containing relevant contrast information) frequencies. BC selectively increases the contrast in the low frequencies within a selectable range of HU. For postprocessing the parameters, center, delta, and slope must be defined. The center describes a window level, delta the range of HU to be processed (comparable to window width). To adapt the contrast intensification, slope can be selected between 0 and 5 (0: CT-images will remain unaffected; 5: maximum contrast enhancement). Comparable to window optimization, center and delta were chosen as mean and twice the SD of the admitted density values. The slope was set at 5.

### 2.6. Reading of Postprocessed NCCT Datasets

Three neuroradiologists (NR 1–3) experienced in acute stroke imaging (NR 1 > 10 years; NR 2/3 > 5 years) independently reviewed all CT datasets (main cohort, control cohorts 1 and 2). Unprocessed NCCT datasets were examined first. With an interval of at least 2 weeks, the NCCT datasets were read again in a varied order supplemented first by the window—and again, at least 2 weeks later, by the BC-optimized CT datasets. The readers had to decide whether an RSSI was present or not. For reading, the original CT request (main collective and control collective 1) and time window (symptom onset to NCCT) were given. For control collective 2, a fictitious CT request (suitable for RSSI) and time specification were randomly assigned. The readers had to mark the infarcted area in case of a positive finding. In addition, a 4-level diagnostic confidence score was recorded: (1) RSSI definitively present; (2) RSSI probably present; (3) RSSI probably not present; (4) RSSI definitively not present. We used this scoring system to represent the radiological reporting habits in clinical routine, where definitive confirmation or exclusion of RSSI may be limited. However, we dichotomized the scoring afterward into RSSI was present (1 and 2) or not (3 and 4). Finally, a true-positive findings required a diagnostic confidence score of 1 or 2 and the correct infarct identification. Correctly marked infarcts with a diagnostic certainty of 3 or 4 were considered as not marked.

### 2.7. Statistical Analysis

We presented categorical variables as absolute and relative frequencies and continuous variables as mean and SD or median and interquartile range (IQR). We used the Kolmogorov–Smirnov test to test for normal distribution. For demographic comparisons, the Mann–Whitney U test was applied. We compared the CT attenuation values and NWU between categorized onset-to-NCCT times using Student’s *t*-test.

The results of blinded reading were evaluated by consensus of the readers. Interrater reliability was calculated using the Fleiss–Kappa function. Control collective 1 was excluded from statistical analysis as the detection of thalamic RSSI was the main objective of the present study. Sensitivity, specificity, and positive/negative predictive values (PPV/NPV) were calculated. To compare sensitivities and specificities, Fisher’s exact test was applied. The significance level was set at α < 0.05.

We performed statistical analyses with SPSS (Version 25; IBM, Armonk, NY, USA) and Excel 2016 (Microsoft Corporation, Redmond, Washington, DC, USA).

## 3. Results

**Figure 2:**
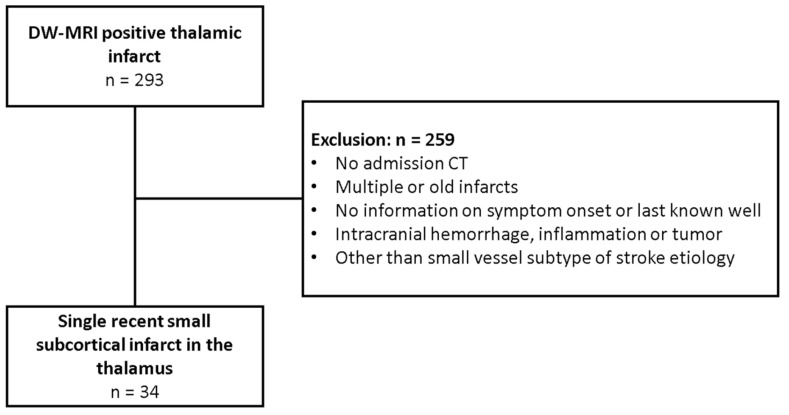
Flow chart of patient selection of the main collective. CT = computed tomography, DW-MRI = diffusion-weighted magnetic resonance imaging.

Of 293 screened patients with thalamic RSSI, 34 met the inclusion criteria (Figure 2). These were 20 men and 14 women with a median age of 70 years (IQR 63–76). The median NIHSS score was 2 (IQR 1–3). Based on the distribution of patients, we arbitrarily categorized the HU and NWU results for thalamic RSSI according to the following time windows: 0–6 h, >6–12 h, >12–24 h, and >24–36 h. The mean time between admission CT and MRI was 2845 min (range 112–14,313). In 23 patients, the RSSI was located in the left thalamus. The mean follow-up infarct DW-MRI volume was 0.35 cm^3^ (±0.3). Further characteristics of patients are summarized in Table 1.

Additionally, 12 and 22 patients were included in the control cohorts 1 and 2, respectively. Age and gender distribution showed no significant differences to the main collective (Appendix A).

All measured CT attenuation values were normally distributed (*p* > 0.05). The mean HU value of RSSI was significantly decreased compared to the unaffected contralateral thalamus (29.6 HU (±3.1) versus 33.3 HU (±2.6); *p* < 0.01). The mean NWU of RSSI was 10.8% (±8) and increased continuously from 6.4% (±7.2) at 0–6 h to 16.6% (±8.7) at 24–36 h from symptom onset or last known well, as well as the CT attenuation values over time (Table 2).

As derived from these results, the optimized window level/width was 30/6 HU, and the optimized BC center/delta was 30/6 HU with a slope of 5. Two of the thalamic RSSIs (6%) were correctly diagnosed on admission CT in routine clinical practice. The NCCT reading in the present study revealed a sensitivity of 32% (11/34) and a specificity of 95% (21/22). In the window- and BC-optimized datasets, sensitivity increased at 38% (13/34) and 41% (14/34), respectively, while specificity decreased to 91% (20/22) and 86% (19/22) (Table 3). Fisher’s exact test showed no significant differences in sensitivity and specificity between unprocessed, window-optimized, and BC-optimized NCCT.

**Figure 3:**
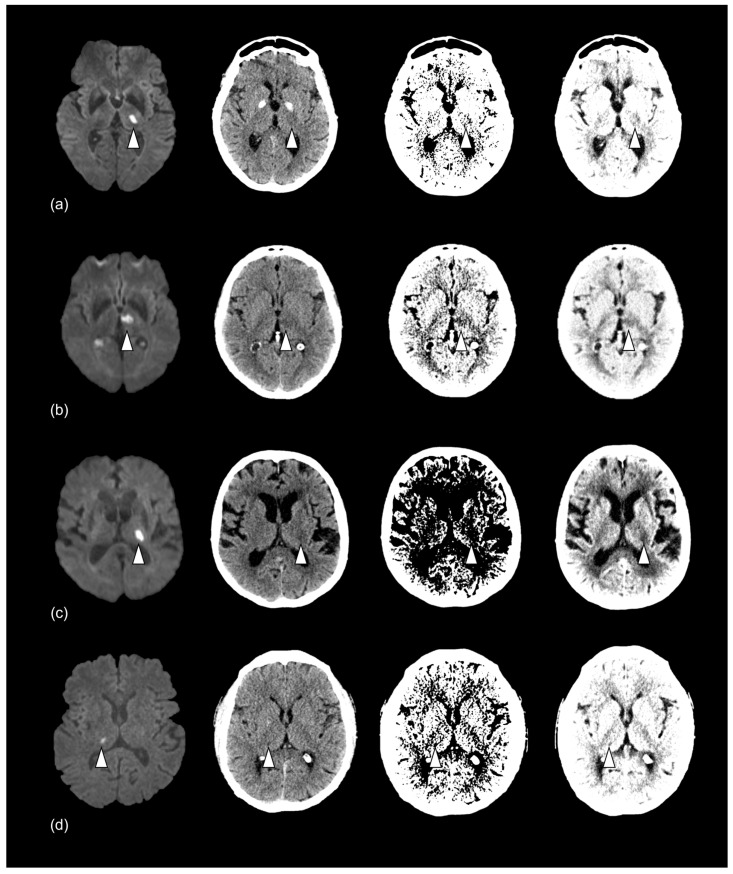
Imaging of thalamic RSSI for the time windows of (**a**) 0–6 h, (**b**) >6–12 h, (**c**) >12–24 h, and (**d**) >24–36 h. Confirmed infarct (white arrowhead) on DW-MRI (left) and corresponding unprocessed, window-, and BC-optimized NCCT (right), respectively. The NWU is 6.2% (**a**), 9.2% (**b**), 15.6% (**c**), and 24.3% (**d**). The infarct was not detected in any NCCT (**a**), detected in window- and BC-optimized NCCT (**b**), only detected in BC-optimized NCCT (**c**), and detected in all NCCT (**d**). RSSI = recent small subcortical infarct, DW-MRI = diffusion-weighted magnetic resonance imaging, BC = Best Contrast, NCCT = non-contrast computed tomography. NWU = net water uptake.

Subdividing these results into the specified time windows showed the lowest sensitivity for RSSI detection 0–6 h after symptom onset (9%). Additional infarcts were correctly diagnosed by adding the window- and BC-optimized CT 6–12 h and 12–24 h after symptom onset (Figure 3; Table 4).

There was moderate agreement between the readers in interpreting the unprocessed NCCT and window-optimized CT datasets (Fleiss kappa = 0.53 and 0.59, respectively). Evaluation of the BC-optimized CT datasets showed fair agreement (Fleiss kappa = 0.4).

## 4. Discussion

Compared with non-affected thalamic tissue, we found a mean decrease of 3.7 HU in RSSI who received NCCT within 36 h after symptom onset. This resulted in a mean NWU of 10.8%, with a continuous increase with extending onset-to-CT times. Image postprocessing based on these results could moderately improve RSSI detection on NCCT.

Data on NWU and CT attenuation of RSSI have not been published in the current literature. Understanding these factors is important as they underlie the neuroimaging correlate of lacunar infarcts, which is ultimately visualized on NCCT. Knowledge of the CT attenuation values over time after symptom onset is, therefore, essential to adapt postprocessing algorithms for improved visualization of these infarcts to the expected HU range. In MCA occlusion, the mean NWU of the ischemic tissue was 9.1% (±6.8) within the first 3.3 h [10]. We found a lower mean NWU of 6.4% (±7.2) within the first 6 h. Concerning CT attenuation, a decrease of 4–5 HU in territorial infarcts within 4–6 h after stroke onset has been reported in monkeys [24]. In rats, even higher density value drops of 7.5–9.6 HU were found within 4–6 h after vascular occlusion [25]. Sufficient data on infarct subtypes other than territorial infarcts are not yet available. In comparison to the reported data, the density decrease of thalamic RSSI is lower. We reported a decrease of 5 HU (±2.5) 12–24 h after symptom onset. The differences could be attributed to the types of tissue involved. Large-vessel occlusion stroke often involves gray and white matter, whereas the thalamus mostly consists of gray matter. Interestingly, the increase in water content in the gray matter of the cortex in experimental MCA occlusion in cats within 4 h was less than 3% [26]. Generalization of our results to other deep gray matter structures, such as the basal ganglia, seems possible and should be investigated in further studies. The application of our findings to RSSI in the deep white matter may be limited because of differences in physiological tissue attenuation and frequent concurrent WML, which may alter the assessment of infarcted and control tissue.

In the present study setting, the correct diagnoses of unprocessed NCCT were 32%. This sensitivity is within the known range of 9–44% for RSSI detection [7,8,9,27]. However, there is a lack of explicit data on thalamic RSSI so far. The limited number of correct diagnoses is presumably primarily due to the slight density differences between the infarcted and surrounding healthy tissue. Image noise is known to mask subtle attenuation decrease [28]. Even advanced imaging with CT perfusion has limited accuracy in detecting RSSI [7,9]. Selective windowing is commonly used to enhance tissue contrast on CT images and to better visualize ischemic infarcts, increasing sensitivity from 57% to 71% in the first 6 h after symptom onset [13]. Another approach of image postprocessing is FSNLB. This method allows a significant improvement of the contrast-to-noise ratio compared to unprocessed NCCT by augmentation of HUs in a predefined range. A previous study showed an increased sensitivity from 54% to 100% in detecting early ischemic edema in acute MCA stroke [12]. Both methods have not yet been evaluated for RSSI diagnostics. Therefore, we adjusted window values and setting parameters of the FSNLB to the previously determined CT attenuation values. Compared to NCCT, sensitivity in the window-optimized and FSNLB-postprocessed datasets increased from 32% to 38% (two additional infarct diagnoses) and 41% (three additional infarct diagnoses), respectively. Thus, the gain in correct diagnoses was lower compared to the windowing and FSNLB in MCA stroke. While FSNLB showed a slight advantage over windowing in our study cohort, both approaches were unable to completely help overcome the subtle attenuation differences that may be masked by image noise. Especially in the early stages of infarction, the reliable diagnosis of RSSI remains restricted to MRI. Particularly where MRI scanner availability is limited, the use of our method could contribute to faster diagnosis of RSSI and timely MRI scans for selected patients. Every additional infarct diagnosis is useful in clinical practice to streamline patient management and therapy. Therefore, causation, rehabilitation, and secondary prevention could be established in the case of early RSSI detection. Image postprocessing can thus be helpful, even though we recorded a small drop in specificity.

The significance of clinical information in RSSI diagnosis should be emphasized. It is noteworthy that in routine clinical practice, only two cases (6%) of RSSI in our study cohort were diagnosed correctly on NCCT. However, with the inclusion of precise clinical information in our study setting, specifically the “clinical suspicion of RSSI”, the correct diagnoses improved to 32%. These results highlight the significant diagnostic benefit obtained by incorporating precise clinical input into the radiological reporting of NCCT datasets, underscoring the importance of close collaboration between neurologists and neuroradiologists.

Recently, commercially available artificial intelligence software showed higher detection rates of early ischemic changes in MCA stroke compared to experienced radiologists [29]. Whether the application of these methods to RSSI is feasible and helps to improve detection rates needs to be evaluated in the future.

A limitation of our study is that different pathophysiological processes of ischemic infarction are visualized on NCCT and MRI in the early stage. DW-MRI delineates the cytotoxic edema, whereas NCCT visualizes the later occurring ionic edema [11]. However, DW-MRI lesions showed corresponding T2-weighted hyperintensities correlating with ionic and vasogenic edema. Second, with regard to the time between NCCT and DW-MRI, it remains unclear whether the infarct size is the same in both modalities. Previous studies indicate a discrepancy in volumes [30]. To minimize the bias from including HU values of unaffected tissue, we reduced the segmented infarct volume before mirroring it onto the NCCT. The CT attenuation values showed a steady decrease over time, so that validity can be assumed. Finally, due to the retrospective study design, the impact of window optimization and FSNLB in everyday clinical practice was not possible. However, it is worth mentioning that our proposed postprocessing techniques are routinely utilized in our clinical practice and can be easily transferred to other hospitals. Window leveling is easy to perform and is part of the daily routine in radiology. To implement the FSNLB postprocessing algorithm (“Best Contrast”), the SyngoVia Client Server application (Research Frontier; Siemens Healthineers, Erlangen, Germany) is required. It is important to emphasize that both postprocessing algorithms are not time-consuming and do not impose any additional burden on the patient.

## 5. Conclusions

In conclusion, we identified CT attenuation values and NWU of RSSI in the thalamus up to 36 h after symptom onset and found NWU increases over time. NCCT postprocessing could increase sensitivity in detecting RSSI moderately, representing a quick and non-harmful tool for improving acute diagnosis when MRI is not available

## Figures and Tables

**Table 1:** Patient characteristics of the main collective.

Variable	
Subjects, No. (%)	34 (100)
Female sex, No. (%)	14 (42)
Age, median (IQR), years	70 (63–76)
NIHSS score, median (IQR)	2 (1–3)
Vascular risk factors, No. (%)	
Hypertension	29 (85)
Hyperlipidemia	22 (65)
Diabetes	16 (47)
Current smoker	4 (12)
Symptom onset (or last known well) to CT, No. (%)	
0–6 h	11 (32)
>6–12 h	7 (21)
>12–24 h	9 (26)
>24–36 h	7 (21)
Admission CT to MRI time, mean (range), min	2845 (112–14,313)
White matter lesions (Fazekas score), No. (%)	
0	3 (9)
1	25 (73)
2	5 (15)
3	1 (3)
Recent small subcortical infarcts in the thalamus	
Side left, No. (%)	23 (68)
Volume at follow-up MRI, mean (SD), cm^3^	0.35 (±0.3)
Hounsfield units (HU), mean (SD)	29.6 (±3.1)
Net water uptake, mean (SD), %	10.8 (±8)
Unaffected contralateral thalamus, HU, mean (SD)	33.3 (±2.6)

CT = computed tomography, HU = Hounsfield units, IQR = interquartile range, MRI = magnetic resonance imaging, NIHSS = National Institutes of Health Stroke Scale, SD = standard deviation.

**Table 2:** Net water uptake and CT attenuation based on onset-to-CT-time.

Time Window	Infarct, Mean (SD), HU	Contralateral Area, Mean (SD), HU	*p*-Value	NWU, Mean (SD), %
0–6 h	31.3 (±2.7)	33.5 (±2.8)	0.016	6.4 (±7.2)
>6–12 h	30.2 (±2.3)	32.6 (±2.6)	0.002	7.3 (±3.4)
>12–24 h	29.5 (±3.3)	34.5 (±2.8)	0.000	14.4 (±7.1)
>24–36 h	26.6 (±2.1)	32.0 (±1.9)	0.004	16.6 (±8.7)

CT = computed tomography, NWU = net water uptake, HU = Hounsfield units, SD = standard deviation.

**Table 3:** Sensitivity, specificity, and predictive values of blinded reading.

	NCCT, %	NCCT + Window-Optimized NCCT, %	NCCT + BC-Optimized NCCT, %
Sensitivity	32	38	41
Specificity	95	91	86
PPV	92	87	82
NPV	48	49	49

NCCT = non-contrast computed tomography, BC = Best Contrast, PPV = positive predictive value, NPV = negative predictive value.

**Table 4:** True positive infarct findings based on onset-to-computed-tomography-time.

Time Window (Symptom Onset—NCCT)	NCCT		NCCT + Window-Optimized NCCT		NCCT + BC-Optimized NCCT	
	Correctly Detected	Not Detected	Correctly Detected	Not Detected	Correctly Detected	Not Detected
0–6 h, No. (%)	1/11 (9)	10/11 (91)	1/11 (9)	10/11 (91)	1/11 (9)	10/11 (91)
>6–12 h, No. (%)	1/7 (14)	6/7 (86)	3/7 (43)	4/7 (57)	3/7 (43)	4/7 (57)
>12–24 h, No. (%)	5/9 (56)	4/9 (44)	5/9 (56)	4/9 (44)	6/9 (67)	3/9 (33)
>24–36 h, No. (%)	4/7 (57)	3/7 (43)	4/7 (57)	3/7 (43)	4/7 (57)	3/7 (43)
Overall, No. (%)	11/34 (32)	23/34 (68)	13/34 (38)	21/34 (62)	14/34 (41)	20/34 (59)

NCCT = non-contrast computed tomography, BC = Best Contrast.

## Data Availability

The datasets generated and analyzed during this study are included in this published article and are available from the corresponding author on reasonable request.

## Appendix A

**Table A1:** Comparison of baseline demographic features of the patient collectives.

	Main Collective	Control Collective 1	Control Collective 2	*p*-Value
Subjects, No. (%)	34 (100)	12 (100)	22 (100)	-
Female, No. (%)	14 (41)	5 (42)	11 (50)	>0.05
Mean age No. (SD), years	69.6 (±8.7)	70.3 (±14.2)	72.1 (±11.4)	>0.05

SD = standard deviation.
